# Supplementary material for: Diversity and Composition of Culturable Microorganisms and Their Biodeterioration Potentials in the Sandstone of Beishiku Temple, China
Source: Microorganisms. 2023 Feb 8;11(2):429. doi: 10.3390/microorganisms11020429 (PMC9965415; doi:10.3390/microorganisms11020429)
Supplement: Supplementary file 1 [file microorganisms-11-00429-s001.zip › microorganisms-2132257-supplementary.pdf]

Supplementary materials for

Microorganisms

Diversity and composition of culturable microorganisms and their biodeterioration potentials on sandstone Beishiku Temple, China

Yong Zhang[1, †], Min Su[1, †], Fasi Wu[1,2,4,*], Ji-Dong Gu[3,5], Jie Li[2,4], Dongpeng He[2,4], Qinglin Guo[2,4], Huiping Cui[2,4], Qi Zhang[1], Huyuan Feng[1,4,*]

[1] *MOE Key Laboratory of Cell Activities and Stress Adaptations, School of Life Sciences, Lanzhou University, Lanzhou, Gansu 730000, The People's Republic of China*

[2] *National Research Center for Conservation of Ancient Wall Paintings and Earthen Sites, Department of Conservation Research, Dunhuang Academy, Dunhuang, Gansu 736200, The People's Republic of China*

[3] *Environmental Science and Engineering Group, Guangdong Technion - Israel Institute of Technology, 241 Daxue Road, Shantou, Guangdong 515063, The People's Republic of China*

[4] *Gansu Provincial Research Center for Conservation of Dunhuang Cultural Heritage, Dunhuang 736200, People's Republic of China*

[5] *Guangdong Provincial Key Laboratory of Materials and Technologies for Energy Conversion, Guangdong Technion - Israel Institute of Technology, 241 Daxue Road, Shantou, Guangdong 515063, The People's Republic of China*

[*] Corresponding author: fenghy@lzu.edu.cn (H. Feng); wufs@dha.ac.cn (F. Wu)

[†] Co-first author, these authors contributed equally to this work;

Table S1. Samples and information of the Beishiku Temple analyzed in this study

**Sample name**	**Merged name**	**Spatial location**	**Sample types**	**Sampling time**	**Height above ground** (m)	**Cave No.**
SD1		Outside	Biofilm	Apr. 2018	0.2	C270
SD2		Outside	Biofilm	Apr. 2018	0.2	C10
SD3	EO-1	Outside	Biofilm	Apr. 2018	0.2	C19
SM1		Outside	Biofilm	Apr. 2018	1.5	C270
SM2		Outside	Biofilm	Apr. 2018	1.5	C10
SM3		Outside	Biofilm	Apr. 2018	1.5	C19
SD4		Outside	Weathered powder	Apr. 2018	0.2	C270
SD5		Outside	Weathered powder	Apr. 2018	0.2	C10
SD6	EO-2	Outside	Weathered powder	Apr. 2018	0.2	C19
SM4		Outside	Weathered powder	Apr. 2018	1.5	C270
SM5		Outside	Weathered powder	Apr. 2018	1.5	C10
SM6		Outside	Weathered powder	Apr. 2018	1.5	C19
SN1		Inside	Biofilm	Apr. 2018	2.3	C9
SN2	SO-1	Inside	Biofilm	Apr. 2018	2.3	C9
SN3		Inside	Biofilm	Apr. 2018	2.3	C9
SN4		Inside	Weathered powder	Apr. 2018	2.3	C9
SN5	SO-2	Inside	Weathered powder	Apr. 2018	2.3	C9
SN6		Inside	Weathered powder	Apr. 2018	2.3	C9
SU1		Outside	Biofilm	Apr. 2018	10.5	C281
SU2	ET-1	Outside	Biofilm	Apr. 2018	10.5	C281
SU3		Outside	Biofilm	Apr. 2018	10.5	C281
SU4		Outside	Weathered powder	Apr. 2018	10.5	C281
SU5	ET-2	Outside	Weathered powder	Apr. 2018	10.5	C281
SU6		Outside	Weathered powder	Apr. 2018	10.5	C281

Note: EO-exposed outside ground; SO-semi-open environment; ET-exposed outside top. 1-Biofilm sample; 2-Weathered powder sample.

Table S2. Physiochemical properties of samples and environmental data of sampling sites

**Sample**	**EO-1**	**EO-2**	**SO-1**	**SO-2**	**ET-1**	**ET-2**	***p***
Cl^-	0.04±0.005b	0.81±0.13a	0.05±0.02b	0.11±0.04b	0.16±0.10b	0.93±0.30a	<0.001
SO_4^{2-}	0.26±0.09b	5.9±0.95a	2.87±1.76ab	2.94±0.74ab	1.12±0.60b	1.61±0.43a	<0.001
NO_3^-	0.36±0.04a	3.35±1.22b	0.19±0.11ab	0.29±0.11ab	0.44±0.21ab	1.77±0.66ab	<0.05
Na^+	0.17±0.03b	4.25±1.36a	0.24±0.03ab	0.28±0.08ab	0.64±0.23ab	3.71±1.19ab	<0.01
K^+	0.74±0.13	5.59±2.33	0.72±0.12	0.5±0.09	0.72±0.12	1.37±0.70	0.092
Mg^{2+}	0.17±0.03	0.64±0.35	0.46±0.04	0.36±0.06	0.35±0.07	0.35±0.04	0.669
Ca^{2+}	3.17±0.26	6.19±1.23	5.94±1.86	4.11±0.85	4.32±1.07	3.46±0.49	0.193
Mc	5.01%±0.62%b	3.31%±0.62%b	2.64%±0.44%b	1.97%±0.11%b	16.49%±7.13%a	6.98%±3.88%b	<0.05
pH	7.64±0.07ab	7.51±0.03b	8.01±0.10a	7.48±0.17b	7.70±0.20ab	7.86±0.09ab	<0.05
Ec	132.58±11.82b	278.17±19.22a	158.47±29.90b	112.60±17.78b	168.03±53.13ab	158.43±29.46b	<0.001
Tem	18.05	17.20	15.81	13.68	17.97	17.44	Ns
RH	56.63	46.21	73.50	89.72	101.32	82.73	Ns

Note: Data are means ± the standard error; Contents of soluble ions are represented by mg per gram (mg/g) of sample; Mc (%) and Ec (us/cm) are short for moisture content and electrical conductivity in physiochemical properties, respectively; Tem (°C) and RH (%) are short for temperature and relative humidity in environmental data, respectively. The different letters indicate the significance of each means in the same row ($p < 0.05$); Significant differences were determined by one-way ANOVA and the *p*-value in the last column.

Table S3. Description of the isolated bacteria

Strain	Bacterial isolates	Accession numbers
bac1	*Staphylococcus epidermidis*	MT076216
bac3	*Microbacterium arthrosphaerae*	MT076217
bac4	*Brevundimonas* sp.	MT076218
bac8	*Microbacterium* sp.	MT076219
bac13	*Lentzea violacea*	MT076258
bac15	*Modestobacter* sp.	MT076245
bac17	*Bacillus subtilis*	MT076221
bac24	*Alphaproteobacteria bacterium*	MT076222
bac29	*Blastococcus saxobsidens*	MT076223
bac32	*Microbacterium phyllosphaerae*	MT076249
bac34	*Nocardioides* sp.	MT076224
bac36	*Roseomonas nepalensis*	MT076225
bac37	*Pseudomonas* sp.	MT076226
bac38	*Sphingomonadaceae bacterium*	MT076250
bac39	*Microbacterium foliorum*	MT076227
bac40	*Sphingomonas* sp.	MT076228
bac41	*Massilia* sp.	MT076229
bac44	*Hymenobacter* sp.	OL854196
bac45	*Nocardioides ganghwensis*	MT076246
bac46	*Arthrobacter* sp.	MT076230
bac61	*Streptomyces* sp.	MT076233
bac65	*Agrococcus citreus*	MT076235
bac67	*Paracoccus* sp.	MT076251
bac69	*Arthrobacter* sp.	MT076252
bac73	*Acinetobacter pittii*	MT076253
bac70	*Kocuria* sp.	MT076257
bac76	*Flavobacterium* sp.	OL854197
bac72	*Bacillus* sp.	MT076236
bac80	*Naxibacter alkalitolerans*	MT076237
bac87	*Arthrobacter* sp.	MT076238
bac89	*Pontibacter* sp.	OL854198
bac91	*Micrococcales bacterium*	MT076239
bac93	*Bacillus* sp.	MT076240
bac96	*Deinococcus* sp.	MT076256
bac98	*Sphingomonas pseudosanguinis*	MT076242
bac99	*Streptomyces* sp.	MT076243
bac100	*Novibacillus thermophilus*	MT076247
bac102	*Bacillus firmus*	MT076244
bac105	*Gemmatimonas* sp.	OL854199

Table S4. Description of the isolated fungi and deteriorative potentials test

**Strain**	**Fungal isolates**	**Accession numbers**	**Pigment secretion**	**Calcite dissolution**
S1	*Engyodontium* sp.	MN982317	-	-
S2	*Muriphaeosphaeria* cfr. *ambrosiae*	MN982318	-	-
S3	*Didymellaceae* sp.	MN982337	-	-
S4	*Coprinellus* cfr. *radians*	MN982343	-	-
S5	*Gibellulopsis* cfr. *nigrescens*	MN982322	-	-
S6	*Cladosporium* cfr. *sphaerospermum*	MN982316	-	+
S8	*Ascomycota* sp.	MN982347	-	-
S9	*Alternaria* cfr. *alternata*	MN982324	-	-
S10	*Cladosporium* cfr. *halotolerans*	MN982327	-	-
S12	*Phoma* sp.	MN982346	-	-
S13	*Rhizopus* cfr. *arrhizus*	MN982339	-	-
S15	*Penicillium* sp.	MN982328	-	+
S17	*Penicillium* cfr. *chrysogenum*	MN982342	+	+
S20	*Ophiobolus* cfr. *artemisiae*	MN982348	+	-
S23	*Chaetomium* cfr. *luteum*	MN982349	+	-
S24	*Cladosporium* sp.	MN982345	-	-
S25	*Cladosporium* cfr. *parahalotolerans*	MN982326	-	-
S26	*Epicoccum* cfr. *nigrum*	MN982330	+	-
S27	*Penicillium* cfr. *citrinum*	MN982335	-	+
S28	*Alternaria* cfr. *compacta*	MN982334	-	-
S29	*Alternaria* cfr. *porri*	MN982333	-	-
S30	*Penicillium* cfr. *rubens*	MN982331	-	+

Table S5. The average inhibition zone of isolated fungi in the antimicrobial test

Strain	The inhibition zone (mm)								
	9-1	9-2	9-3	9-4	9-5	9-6	9-7	9-8	9-9
S1	10	12	6	6	10	12	10	10	9
S2	9	7	6	6	10	11	8	10	14
S5	8	12	8	6	6	9	8	10	11
S6	10	12	8	8	12	16	10	10	11
S8	10	12	6	12	6	11	6	11	10
S9	18	12	8	10	8	12	8	8	10
S10	16	18	6	8	13	18	10	8	12
S12	15	9	6	12	10	13	8	10	10
S13	12	11	16	8	10	18	14	12	14
S15	10	10	6	8	15	13	10	10	8
S17	12	8	12	8	14	12	12	14	18
S20	11	6	14	10	7	12	11	8	8
S23	9	18	10	9	8	12	11	6	10
S24	12	12	14	9	6	10	10	7	12
S25	10	16	8	13	8	13	10	8	10
S26	16	12	6	6	6	12	11	10	16
S27	12	12	8	8	10	15	8	8	10
S28	12	20	9	8	9	8	6	6	8
S29	10	9	10	6	10	14	8	10	17
S30	14	12	8	8	8	12	8	8	10
Maximum	18	20	16	13	15	18	14	14	18
Minimum	8	6	6	6	6	6	6	6	8
Mean	11.8	12	8.75	8.45	9.3	12.65	9.35	9.2	11.4

Note: Different numbers represent different biocides. 9-1: Dichlorophen; 9-2: 1,2-Benzisothiazolin-3-One; 9-3: Didecyldimethylammonium Chloride; 9-4: Benzalkonium Chloride; 9-5: Isothiazolinones; 9-6: 2-Octyl-2H-isothiazol-3-one; 9-7: Natamycin; 9-8: 2-Butyl-1,2-benzisothiazolin-3-one; 9-9: 4,5-Dichloro-2-octyl-isothiazolone

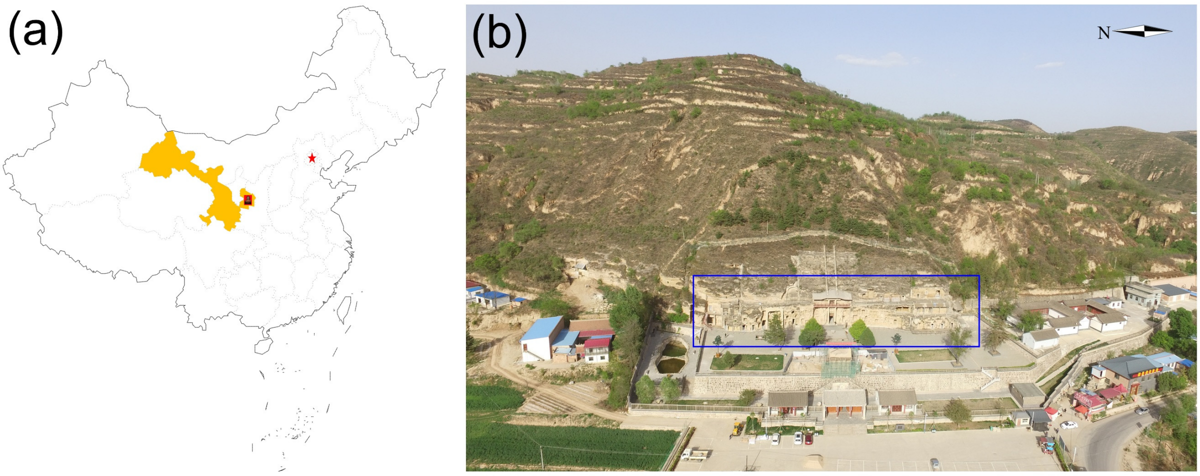

Figure S1. Location of the study site. a: location of the Beishiku Temple in China; b: aerial image of the site (blue frame) with nearby surroundings.

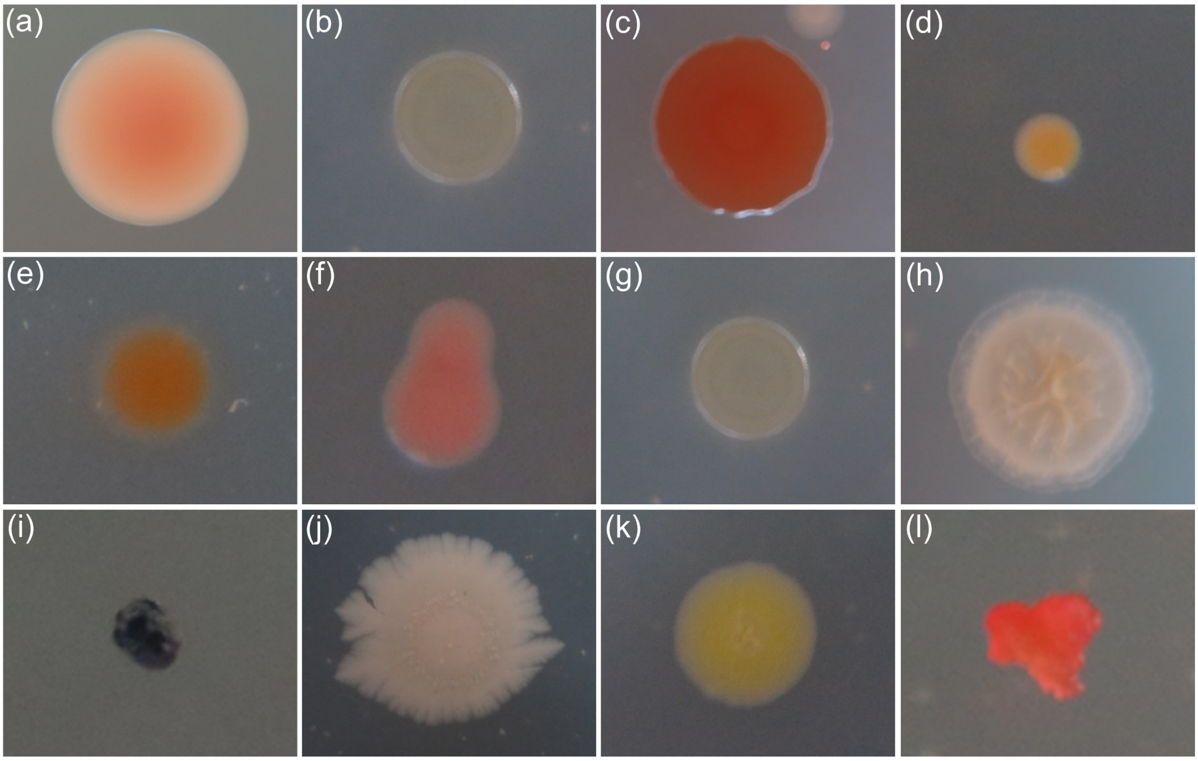

Figure S2. The morphological appearance of the representative culturable bacterial strains. (a): *Staphylococcus* sp.; (b): *Massilia* sp.; (c): *Sphingomonadaceae bacterium*; (d): *Arthrobacter* sp.; (e): *Paracoccus* sp.; (f): *Roseomonas* sp.; (g): *Pseudomonas* sp.; (h): *Bacillus* sp.; (i): *Brevundimonas* sp.; (j): *Sphingomonas* sp.; (k): *Arthrobacter* sp.; (l): *Sphingosinicella* sp.

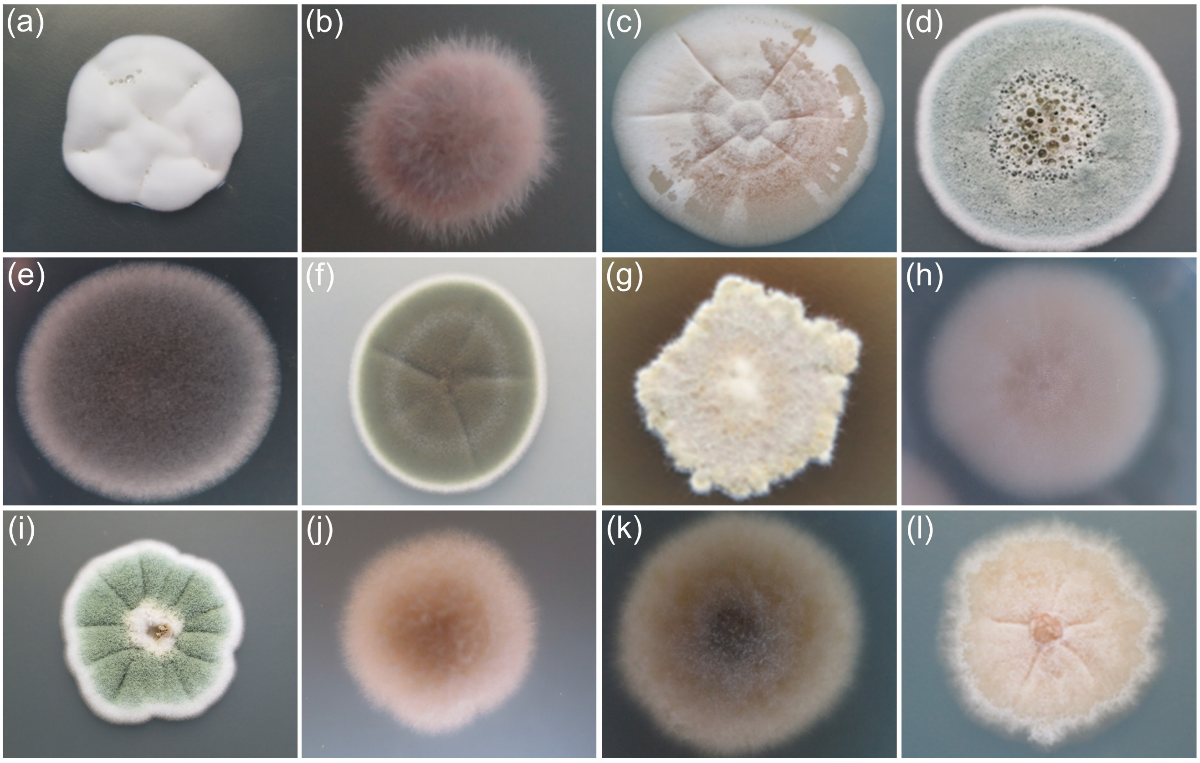

Figure S3. The morphological appearance of the representative culturable fungal strains. (a): *Engyodontium* sp; (b): *Alternaria* sp.; (c): *Coprinellus* cfr. *radians*; (d): *Penicillium* sp.; (e): *Cladosporium* cfr. *parahalotolerans*; (f): *Cladosporium* cfr. *sphaerospermum*; (g): *Epicoccum* cfr. *nigrum*; (h): *Ascomycota* sp.; (i): *Penicillium* cfr. *citrinum*; (j): *Gibellulopsis* cfr. *nigrescens*; (k): *Muriphaeosphaeria* cfr. *ambrosiae*; (l): *Phoma* sp.

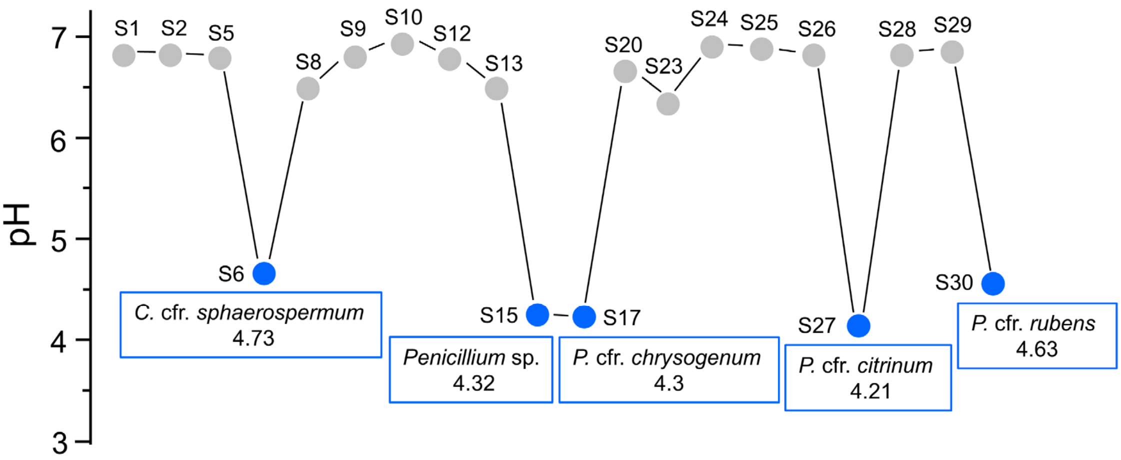

Figure S4. Variation of pH in broth cultures of tested fungal isolates (day 7, Czapek-Dox liquid medium). Each dot on the chart represents one of the isolated fungi.

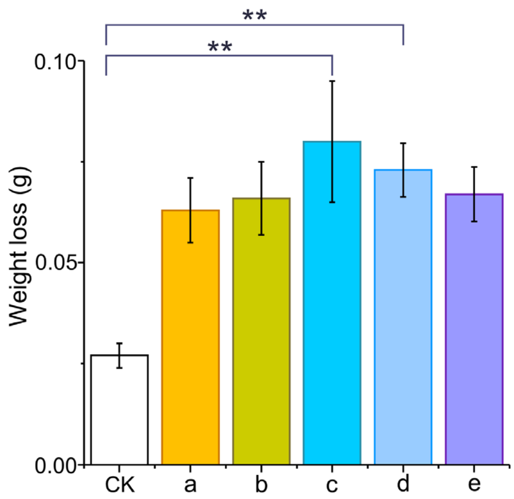

Figure S5. Weight loss of sandstone blocks cultured with acidogenic fungi in the Czapek-Dox liquid medium. (CK) the Czapek-Dox liquid medium; (a) *P.* cfr. *rubens*; (b) *P.* cfr. *chrysogenum*; (c) *P.* cfr. *citrinum*; (d) *C.* cfr. *sphaerospermum*; (e) *Penicillium* sp. Significant results were indicated with asterisks (*). Significance levels (p): "**" $p < 0.01$.

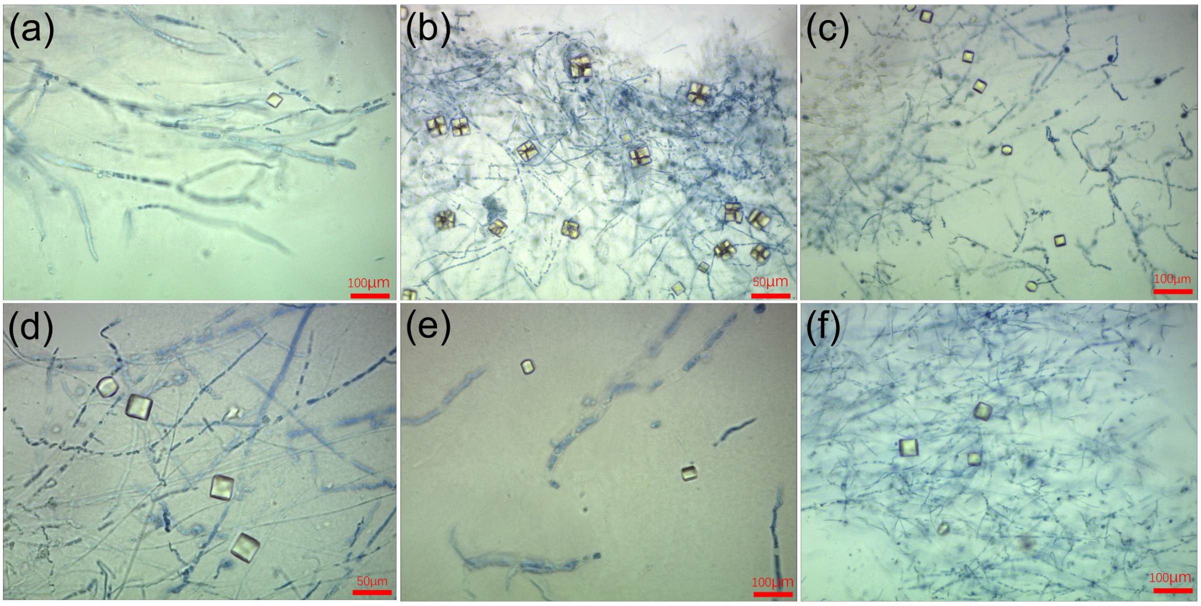

Figure S6. Optical microscope observation of the precipitated crystals from fungi. (a) *P.* cfr. *rubens*; (b) *P.* cfr. *chrysogenum*; (c) *P.* cfr. *citrinum*; (d, e) *C.* cfr. *sphaerospermum*; (f) *Penicillium* sp.

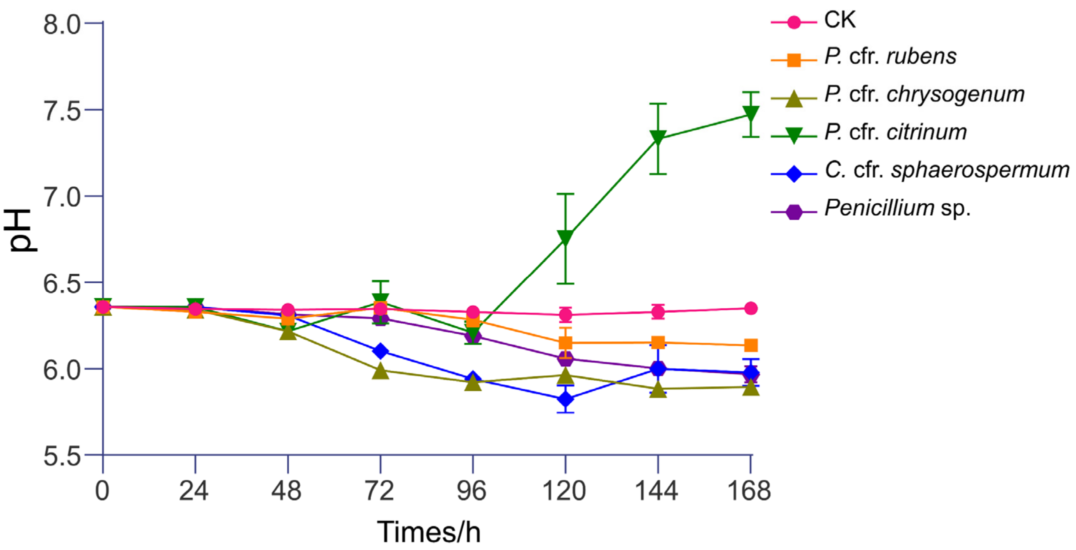

Figure S7. pH of the liquid MB4 medium with different fungal strains. (CK) the liquid MB4 medium without fungal strains.
